# Phosphorylated septin 3 delocalizes from the spine base and facilitates endoplasmic reticulum extension into spines via myosin-Va

**DOI:** 10.1186/s13041-025-01215-9

**Published:** 2025-05-15

**Authors:** Natsumi Ageta-Ishihara, Masato Mizukami, Itsuki Kinoshita, Yurika Asami, Tomoki Nishioka, Haruhiko Bito, Kozo Kaibuchi, Makoto Kinoshita

**Affiliations:** 1 Department of Biomolecular Science, Faculty of Science, Toho University, 2-2-1 Miyama, Funabashi, 274-8510 Chiba Japan; 2 Department of Molecular Biology, Division of Biological Sciences, Graduate School of Science, Nagoya University, Chikusa-ku, Nagoya, 464-8602 Japan; 3 Division of Cell Biology, International Center for Brain Science, Fujita Health University, Toyoake, 470-1192 Aichi Japan; 4 Open Facility Center, Research Promotion Headquarters, Fujita Health University, Toyoake, 470-1192 Aichi Japan; 5 Department of Neurochemistry, Graduate School of Medicine, The University of Tokyo, Bunkyo-ku, Tokyo, 113-0033 Japan

**Keywords:** Septin, Phosphorylation, Smooth Endoplasmic reticulum, Spine

## Abstract

Cytoskeletal remodeling drives morphological changes. Septin cytoskeleton assembles into hetero-oligomers. We previously demonstrated that late-phase long-term potentiation (L-LTP) induces smooth endoplasmic reticulum (sER) extension into dendritic spines via septin 3 (SEPT3), contributing to greater postsynaptic Ca^2+^ responses and enhanced activation of synaptically induced Ca^2+^ signaling. *Sept3*^−/−^ mice exhibit a reduced number of sER-containing spines and show impaired long-term spatial/object memory despite normal short-term memory. Additionally, SEPT3 binds the motor protein myosin-Va (MYO5A) upon elevated Ca²⁺ concentrations, facilitating sER extension from the dendritic shaft into the spine. MYO5A localizes on the sER membrane, while SEPT3 remains at the spine base, accumulating on sER upon electroconvulsive stimulation (ECS). However, the mechanism underlying SEPT3’s delocalization from the spine base and its cooperative role with MYO5A in sER extension remains unclear. In this study, we demonstrate that SEPT3 is phosphorylated in a stimulation-dependent manner. Phosphorylation at Thr211 releases SEPT3 from the spine base, enabling sER extension with constitutively active MYO5A mutant (MYO5A-CCtr). These findings provide molecular insight into the role of SEPT3 phosphorylation in regulating sER dynamics that sustain long-term spine activation.

**Supplementary Information:**

The online version contains supplementary material available at 10.1186/s13041-025-01215-9.

## Main text

L-LTP-dependent actin reorganization underlies structural plasticity [1–3], but mechanisms maintaining prolonged synaptic activation in enlarged spines remain unclear. We previously showed that L-LTP induces sER extension into dentate gyrus (DG) granule cell spines via SEPT3, supporting sustained Ca²⁺ responses and synaptic activation. Furthermore, *Sept3*^−/−^ mice [4], with fewer sER-containing spines, show normal short-term but impaired long-term spatial memory, with the hippocampal DG as the responsible region. We showed that sER extension is regulated by the interaction between the activated MYO5A, which undergoes Ca^2+^-triggered conformational changes, and SEPT3. Additionally, we demonstrated that part of MYO5A localizes on the sER membrane, whereas SEPT3 is not under basal conditions. However, while strong stimulation induces SEPT3 accumulation on the sER membrane [5], the mechanism underlying this process remains unknown.

**Fig. 1:**
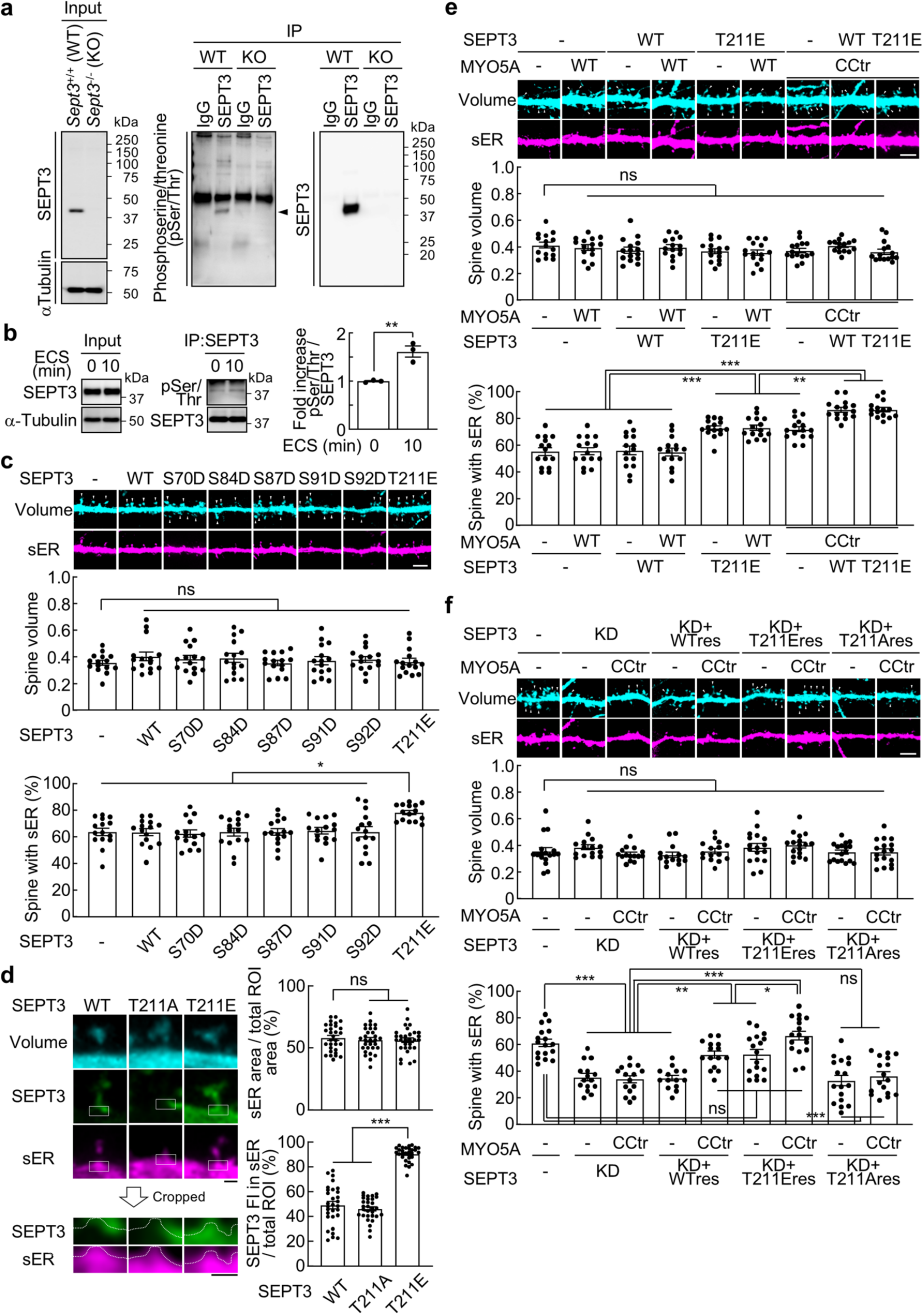
Phosphorylation of Thr211 residue of SEPT3 delocalizes it from the dendritic spine base and promotes sER extension into spines in cooperation with activated MYO5A **a**, Co-IP with IgG or anti-SEPT3 antibodies using protein extracted from the hippocampal DG of *Sept3*^+/+^ and *Sept3*^−/−^ male mice, followed by IB analysis using antibodies against SEPT3, α-Tubulin, and phosphoserine/threonine (pSer/Thr). Arrowhead indicates the SEPT3 signal. **b**, Left and middle, Co-IP with anti-SEPT3 antibody using protein extracted from the hippocampal DG of *Sept3*^+/+^ male mice at 0 min and 10 min after ECS. Right, Normalized phosphorylation level of SEPT3 on Ser/Thr. *n* = 3 experiments; two-tailed unpaired *t* test. **c**, Top, Representative images of dendritic spines and sER in primary cultured rat DG granule cells expressing wild-type (WT) or phosphomimetic mutants of SEPT3, showing the morphology of spines (cyan) and sER (magenta) at 21 days *in vitro* (DIV). Scale bar, 5 μm. Middle, Spine volume, defined as the total fluorescence intensity of the spine within regions of interest (ROIs) divided by the average fluorescence intensity of the dendrite within ROIs. Bottom, Percentage of spines with sER. *n* = 15 dendrites; one-way ANOVA with Tukey’s multiple comparisons test. **d**, Left, Representative images of dendritic spines and GFP-tagged SETP3-WT, non-phosphorylatable alanine substitution mutant (T211A), or phosphomimetic aspartic acid mutant (T211E). White squares indicate the regions used for analysis. In the magnified images, white dashed lines outline the boundary between the cytosol and sER. Scale bar, 0.5 μm. Right top, Quantification of sER area as a percentage of the total region of interest (ROI). Right bottom, Quantification of the proportion of GFP-SEPT3 fluorescence intensity (IF) within the sER area relative to the total ROI. *n* = 30 spines; one-way ANOVA with Tukey’s multiple comparisons test. **e**, Top, Representative images of dendritic spines and sER from DG granule cells overexpressing SEPT3-WT or T211E, transiently co-expressing either MYO5A-WT or CCtr (the functionally active form). Scale bar, 5 μm. Middle, Spine volume. Bottom, Percentage of spines with sER. *n* = 15 dendrites; one-way ANOVA with Tukey’s multiple comparisons test. **f**, Top, Representative images of dendritic spines and sER from DG granule cells expressing shSEPT3, an shRNA resistant mutant of SETP3-WT, T211E, or T211A, and/or co-expressing MYO5A-WT or CCtr. Scale bar, 5 μm. Middle, Spine volume. Bottom, Percentage of spines with sER. *n* = 14–17 dendrites; one-way ANOVA with Tukey’s multiple comparisons test. Data are mean ± s.e.m. *p < 0.05, **p < 0.01, ***p < 0.001, ns, not significantly different.

Post-translational modifications regulate protein localization, and given that many signaling molecules undergo phosphorylation upon LTP induction in the hippocampal CA1 region [6], phosphorylation plays a crucial role in synaptic plasticity. Therefore, we investigated whether SEPT3 is phosphorylated in response to strong stimulation in the hippocampus DG region. Following our previous study [5], we performed co-immunoprecipitation (co-IP) with immunoglobulin G (IgG) or SEPT3 antibodies using proteins extracted from the hippocampal DG of 9-week-old male *Sept3*^+/+^ or *Sept3*^−/−^ mice. Immunoblot (IB) analysis using a phosphoserine/threonine (pSer/Thr) antibody revealed that a band was detected at the predicted molecular weight for SEPT3 only in samples from the DG of *Sept3*^+/+^ mice and co-immunoprecipitated with a SEPT3 antibody (Fig. 1a). In our previous study, we reported that ECS of the molecular layer of the hippocampal DG increased the number of sER-containing spines in *Sept3*^+/+^ mice, but not in *Sept3*^−/−^ mice, and that the association between SEPT3 and MYO5A was increased 10 min after ECS induction [5]. Consistent with previous findings [5], ECS in 8- to 9-week-old *Sept3*^+/+^ male mice increased the pSer/Thr signal at the SEPT3-predicted molecular weight via co-IP. We found that ECS induction increased the pSer/Thr signal detected by the pSer/Thr antibody (Fig. 1b).

Next, to determine the Ser/Thr residues of SEPT3 that are phosphorylated, we predicted the phosphorylation sites of SEPT3 using the PhosphoSitePlus database, identifying Ser70, Ser84, Ser87, Ser91, and Ser92 as candidates. It has been reported that the Ser91 is phosphorylated by PKG, and this phosphorylation is detected in the cytoplasmic fraction of synaptosomes [7, 8]. Furthermore, because phosphorylation of SEPT7 and SEPT12 has been reported to inhibit septin filament formation [9, 10], Thr211 residue, a homologous site in SEPT3, was also added as a candidate. We created these pseudophosphorylation mutants and counted the number of sER-containing spines in primary cultures of hippocampal DG granule cells, where we previously reported L-LTP-dependent sER extension into spines [5]. As a result, only the SEPT3 phosphomimetic aspartic acid mutant (T211E) induced an increase in the number of sER-containing spines with no effect on spine volume (Fig. 1c). These results indicate that Thr211 phosphorylation of SEPT3 facilitates sER extension into spines without affecting spine volume, consistent with our previous findings that SEPT3 regulates sER dynamics independent of spine size [5].

Under basal conditions, SEPT3 is localized at the spine base, but its localization on the sER membrane is increased by ECS induction [5]. We therefore examined the effect of phosphorylation of Thr211 residue of SEPT3 on the localization of SEPT3 by overexpressing GFP-tagged SEPT3-wild-type (WT), non-phosphorylatable alanine substitution mutant (T211A), or phosphomimetic aspartic acid mutant (T211E). SEPT3-WT and T211A localized to the spine base, while T211E showed a higher proportion of signal within the sER region (Fig. 1d).

We hypothesized that phosphorylated SEPT3 and Ca²⁺-activated MYO5A cooperate in sER extension and tested this using SEPT3-T211E and MYO5A-CCtr, which lacks the autoinhibitory globular tail domain of MYO5A and mimics the Ca²⁺-dependent conformational change that activates the motor [11]. As a result, there was no change in spine volume in either case, but the number of sER-containing spines increased upon expression of SEPT3-T211E (Fig. 1e). As in our previous report, co-expression of SEPT3-WT and MYO5A-CCtr further increased the number of sER-containing spines [5], but co-expression of SEPT3-T211E and MYO5A-CCtr caused a similar increase (Fig. 1e). Since the number of sER-containing spines increased by approximately 90% upon chemical L-LTP induction [5], it was predicted that the number of sER-containing spines had reached an upper limit with co-expression of SEPT3-WT and MYO5A-CCtr. Therefore, we examined the cooperation between SEPT3 and MYO5A in extending sER into spines by combining SEPT3 depletion via RNAi. The knockdown efficiency and the expression levels of shRNA-resistant SEPT3 in the rescue experiments were validated by quantitative analysis of immunofluorescence intensity (Fig. S1a, b). SEPT3 deficiency reduced sER-containing spines, partially rescued by shRNA-resistant SEPT3-WT or T211E with MYO5A-CCtr, and fully rescued by T211E with MYO5A-CCtr. In addition, co-expression of shRNA-resistant SEPT3-T211A with MYO5A-CCtr did not rescue it (Fig. 1f). The increase in sER-containing spines by SEPT3-T211E was suppressed by dominant-negative MYO5A (Fig. S2), indicating that both phosphorylated SEPT3 and activated MYO5A are necessary for sER extension. Finally, we tested whether Thr211 phosphorylation enhances SEPT3–MYO5A association. Co-IP using cell lysate co-expressing MYO5A and SEPT3-WT or T211E showed that SEPT3-T211E had greater association with MYO5A than SEPT3-WT (Fig. S3a, b). Previously, we found that SEPT7, a core subunit of the septin complex, localizes to the spine base [12] and remains unchanged on sER localization upon ECS [5]. Together, these results suggest that, following L-LTP, phosphorylation of Thr211 residue of SEPT3 dissociates it from septin complexes at the spine base and interacts with activated MYO5A to regulate the extension of sER into spines.

In the future, if technology can be developed to utilize these molecular mechanisms to induce sER extension into spines, it could enable the artificial induction of long-term memory.

## Methods

Methods are described in the Supplementary Materials.

## Electronic supplementary material

Below is the link to the electronic supplementary material.

Supplementary Material 1

## Data Availability

No datasets were generated or analysed during the current study.

## Abbreviations

L-LTP: Late-phase long-term potentiation
sER: Smooth endoplasmic reticulum
SEPT3: Septin 3
MYO5A: Myosin-Va
ECS: Electroconvulsive stimulation
DG: Dentate gyrus
Co-IP: Co-immunoprecipitation
IgG: Immunoglobulin G
IB: Immunoblot
pSer/Thr: Phosphoserine/threonine
WT: Wild-type
DIV: Days *in vitro*
ROI: Region of interest
