# Supplementary material for: Phosphorylated septin 3 delocalizes from the spine base and facilitates endoplasmic reticulum extension into spines via myosin-Va
Source: Mol Brain. 2025 May 15;18:43. doi: 10.1186/s13041-025-01215-9 (PMC12079886; doi:10.1186/s13041-025-01215-9)
Supplement: Supplementary file 1 — Supplementary Material 1 [file 13041_2025_1215_MOESM1_ESM.pdf]

Supplementary Materials

Methods

Animal experiments

All animal procedures were reviewed and approved by the appropriate institutional ethics committees, and were performed in compliance with the animal care and use guidelines of Nagoya University and Toho University. Wistar ST rats were obtained from Japan SLC. Mice were maintained under standard conditions with a 12-hour light/dark cycle and provided with food and water ad libitum. 8- to 9-week-old *Sept3*$^{-/-}$ male mice [1, 2] and wild-type littermates were used in all experiments.

Antibodies

We used anti-SEPT3 antibody as previously described [1], as well as the following commercial antibodies: anti-α-tubulin (T9026, Sigma, 1:1000), anti-phosphoserine/threonine (pSer/Thr) antibody (612548, BD, 1:1000), anti-FLAG (F3165, Sigma, 1:1000), and Alexa 555- or horseradish peroxidase-conjugated secondary antibodies (A21429, Thermo 7076; 7074, Cell Signaling; 18-8817-31 and 18-8816-33, Rockland; all used at 1:1000).

Plasmid constructs

Most of the plasmids used in this study were previously described [1]. The validation of shRNA knockdown efficiency using *in vivo* AAV infection followed by immunoblotting was also reported in the same study. SEPT3 mutants were generated by site-directed mutagenesis using plasmids in which SEPT3 was expressed under the control of the alpha-CaMKII promoter in a pEGFP- or ECFP-C1–based backbone. All constructs were verified by sequencing.

Immunoprecipitation and immunoblot analysis

Electroconvulsive stimulation (ECS), along with immunoprecipitation and immunoblot analyses, was conducted as previously reported [1, 3]. In brief, ECS was applied via ear clips using an ECT Unit (Model 57800, UGO Basile), delivering a 0.2 sec, 100-Hz, 40-mA stimulus of a 0.2 msec square wave pulse. Animals typically recovered within 1–2 minutes post-stimulation. The dentate gyrus (DG) of the hippocampus was isolated from 8- to 9-week-old male mice, weighed, and homogenized in ice-cold lysis buffer containing 20 mM Tris-HCl (pH 8.0), 150 mM NaCl, 1% Triton X-100, 10% glycerol, 1 mM Na_3VO_4, 20 mM β-Glycerophosphate, 50 mM NaF, phosphatase inhibitor cocktail III (P0044, Sigma), protease Inhibitor (11836153001, Roche). After centrifugation at 20,400 × g for 15 min at 4°C, the supernatant was incubated overnight at 4°C with 2 μg of either anti-SEPT3 antibody or control

rabbit IgG (Cell Signaling, 2729S), along with 40 μL of Protein A Sepharose CL-4B (17-0780-01, GE healthcare). Bound complexes were washed and eluted by boiling in 2 × sample buffer (100 mM Tris-HCl (pH 6.8), 4% SDS, 20% glycerol, 0.01% bromophenol blue, 10% 2-mercaptoethanol) for 5 min. For *in vitro* assays, COS-7 cells (JCRB9127) were maintained in Dulbecco's modified Eagle's medium with 10% FBS and transfected using Lipofectamine 2000 (11668-030, Thermo). After 48 h, cells were lysed using a buffer containing 100 mM Tris-HCl (pH 7.35), 150 mM NaCl, 0.1% SDS, 0.1% Triton X-100, 0.1% sodium deoxycholate, 10 mM 2-mercaptoethanol, and protease inhibitors. Lysates were centrifuged, and the resulting supernatant was incubated with 2 μg of anti-FLAG antibody (F3165, Sigma-Aldrich) and 13 μL of Affi-Prep Protein A Support (1560005, Bio-Rad) for immunoprecipitation.

Morphometric analyses and immunocytochemistry

The preparation of primary hippocampal DG granule cells, as well as morphometric analyses and immunocytochemistry were performed following previously described protocols [1]. In brief, DG granule cells were isolated from postnatal day 1 (P1) Sprague-Dawley rat pups (male and female; SLC) and plated onto poly-D-Lysine-coated glass-bottom 35 mm dishes (P35GC-1.5-14-C, Matteck) at a density of 5 × 10^4 cells per dish. For experiments using the Tet-on system, neurons were treated with 1 μg/ml doxycycline (B-0801, Echelon) for 24 h at DIV 18–22 before imaging. For morphometric analyses, cultured DG granule cells were fixed with 4% paraformaldehyde (PFA)/4% sucrose in phosphate-buffered saline (PBS). For immunocytochemistry, cells were blocked for 1 h at room temperature (RT) in PBS containing 0.1% Triton-X-100, 2% bovine serum albumin, and 8% normal goat serum. Cultured neurons were incubated with primary antibodies diluted in blocking solution for 24 h at 4°C, followed by secondary antibodies for 1 h at RT. Cells were mounted with Aqua-Poly/Mount (18606-20, Polysciences). Fluorescence images were acquired using confocal microscopes (LSM-780 and LSM-900, Zeiss; FV3000, Olympus).

Quantitative image analysis was performed as described previously [1]. Briefly, spine volume was measured using the fluorescence signal of the volume marker (iRFP670), and sER was visualized by expressing pCaMKII:ER-TagRFP. Z-projected xy images were generated in Fiji (ImageJ) by summing pixel intensities. After background subtraction, the percentage of spines containing sER was calculated within a defined region of interest (ROI; 50.71–106.7 μm × 19.81 μm). Spine volume was estimated as the fluorescence intensity ratio between the spine and adjacent dendritic shaft. To quantify the signal of EGFP-SEPT3-WT, T211A, and T211E in the sER region, ROIs were defined using Fiji (ImageJ) for each spine exhibiting GFP-SEPT3 signal at the spine base exceeding twice the background intensity. The ROIs were centered at the spine neck/base boundary and extended 460 nm

laterally and 460 nm from the spine base into the dendritic shaft. Within each ROI, the sER-positive area was manually outlined based on the ER-TagRFP signal. The percentage of EGFP-SEPT3 localized to the sER area was calculated as follows: EGFP-SEPT3 in sER (%) = (sum of EGFP-SEPT3 intensity within sER area in each confocal section) / (sum of total EGFP-SEPT3 intensity within ROI in each confocal section) × 100. To calculate the sER area relative to the total ROI area: sER area (%) = (sum of sER area in each ROI in each section) / (sum of ROI area in each section) × 100. To quantify SEPT3 knockdown efficiency by immunocytochemistry, ROIs were manually defined in the soma of transfected neurons based on the iRFP670 signal. The fluorescence intensity of SEPT3 immunostaining within each ROI was measured for every confocal section, and the values were summed across all sections.

Quantification and statistical analysis

All quantitative results are presented as mean ± SEM (standard error of the mean) or median. Statistical analyses were performed using Prism software (GraphPad). Specific details regarding statistical tests, P values, sample sizes, and error bar definitions are provided in the respective figure legends.

References

1. Ageta-Ishihara N, Fukazawa Y, Arima-Yoshida F, Okuno H, Ishii Y, Takao K, Konno K, Fujishima K, Ageta H, Hioki H, et al: **Septin 3 regulates memory and L-LTP-dependent extension of endoplasmic reticulum into spines.** *Cell Reports* 2025.
2. Fujishima K, Kiyonari H, Kurisu J, Hirano T, Kengaku M: **Targeted disruption of Sept3, a heteromeric assembly partner of Sept5 and Sept7 in axons, has no effect on developing CNS neurons.** *J Neurochem* 2007, **102:**77-92.
3. Cole AJ, Abu-Shakra S, Saffen DW, Baraban JM, Worley PF: **Rapid rise in transcription factor mRNAs in rat brain after electroshock-induced seizures.** *J Neurochem* 1990, **55:**1920-1927.

Supplementary Figures

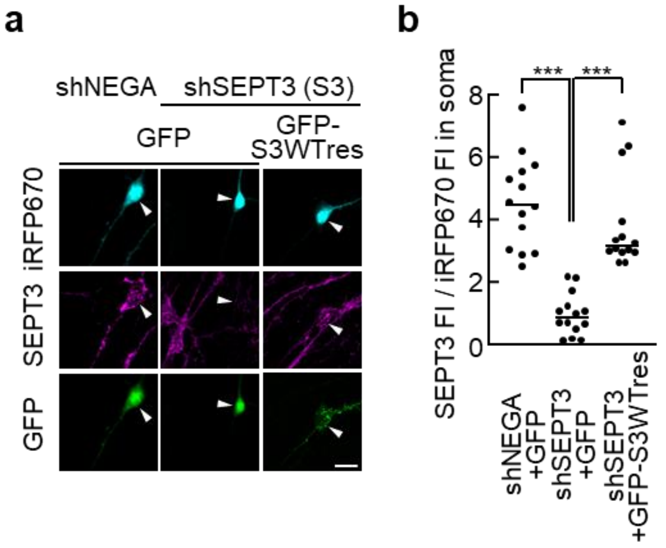

Figure S1

Validation of knockdown and rescue efficiency of SEPT3 using immunocytochemistry.

a, Representative images of cultured hippocampal DG granule cells transfected with control shRNA (shNEGA-iRFP670), SEPT3 knockdown (shSEPT3-iRFP670), or shSEPT3 together with either a mock construct (GFP) or a shRNA-resistant mutant of SEPT3 (GFP-SEPT3-WTres). Cells were stained with anti-SEPT3 antibody (magenta). Arrowheads indicate transfected neurons. Scale bar, 20 μm.

b, Quantification of SEPT3 fluorescence intensity normalized to iRFP670 fluorescence in the soma of transfected neurons. n = 14 neurons; Kruskal-Wallis test with Dunn's multiple comparisons test. Data are median. ***$p < 0.001$.

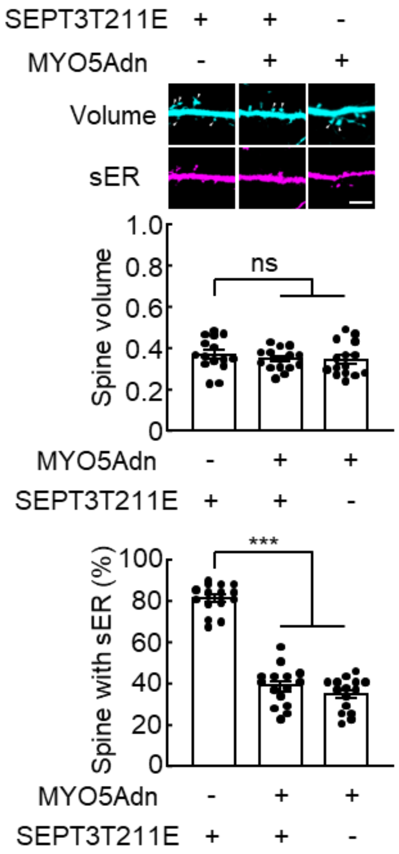

Figure S2

Dominant-negative MYO5A suppresses the increase in the number of sER-containing spines induced by SEPT3-T211E.

Top, Representative images of hippocampal DG granule cells expressing SEPT3-T211E alone, SEPT3-T211E together with dominant-negative MYO5A (MYO5Adn), or expressing MYO5Adn alone. Scale bar, 5 µm. Middle, Spine volume. Bottom, Quantification of the number of sER-containing spines. n = 15 dendrites; one-way ANOVA with Tukey's multiple comparisons test.

Data are mean ± s.e.m. ***$p < 0.001$; ns, not significant.

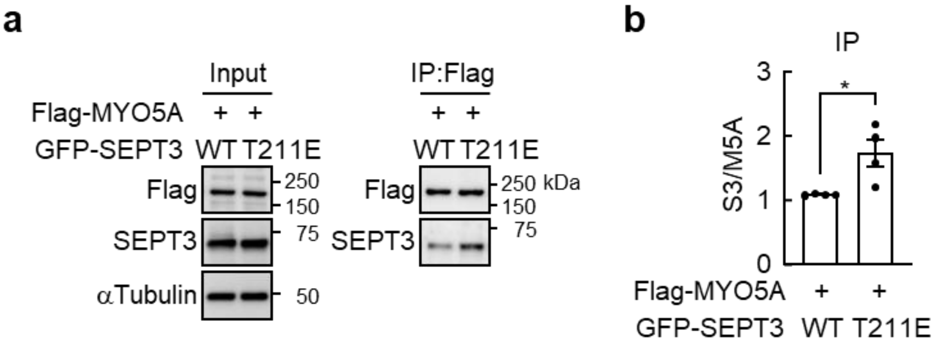

Figure S3

Phosphomimetic SEPT3-T211E enhances association with MYO5A.

a, Co-IP with anti-FLAG antibody using cell lysates expressing Flag-tagged MYO5A and GFP-tagged SEPT3-WT or T211E, followed by IB analysis using antibodies against Flag, SEPT3, and α-Tubulin.

b, Densitometric quantification of the fraction of SEPT3-WT or T211E bound to MYO5A. n=4 experiments; two-tailed unpaired *t* test.

Data are mean ± s.e.m. $*p < 0.05$.
